# Stock-Farm Veterinary Practice as a Post-Graduate Course1Read before the annual meeting of the Pennsylvania State Veterinary Medical Association, March, 1899.

**Published:** 1899-06

**Authors:** A. M. Lushington

**Affiliations:** Lynchburg, Va.


### THE JOURNAL

OF

### COMPARATIVE MEDICINE AND VETERINARY ARCHIVES.

VOL. XX. *JUNE, 1899.* No. 6

### STOCK-FARM VETERINARY PRACTICE AS A POST-GRADUATE COURSE.[1]

BY A. M. LUSHINGTON, V.M.D.,
LYNCHBURG, VA.

1. *Study of Animal Life on the Farm.* The study of animal life on the farm is both interesting and instructive. The conditions under which large numbers of one or more species of domestic animals may be kept on the farm more nearly approximates that to which they are accustomed in nature, and permits of the widest latitude for the display and exhibition of those peculiar instinctive traits which form a part of their distinguishing characteristics. With apparently a keen sense for observation and selection, the members of each species or family readily seek out and crave the association of other members of the same species or family, and appear perfectly satisfied only when thus associated. Wandering at large and grazing through the pastures, or at the drinking-pond or stream, they manifest a deep concern for each other, clearly evidencing, however, that the instinct for association is peculiar to them all. They frisk, frolic, and gambol with a spirit of delight and gratification seemingly peculiar only to themselves. When, for reasons of disability, any member or members refuse to share in a general jollification, the very deepest concern is manifested for him. When danger, real or suspected, threatens and an asylum becomes necessary, as by magic the signal is given, and the course taken by one seems to be the choice of all, and this persistently and unswervingly followed even when conditions of a most threatening and dangerous character sometimes seem impossible to be overcome.

2. *Stock-farm Conditions Simulate a Natural Condition.* The

[1] Read before the annual meeting of the Pennsylvania State Veterinary Medical Association, March, 1899.

conditions under which animals live in nature have a direct similarity to those on the stock-farm. In the former they enjoy so-called absolute and unrestricted freedom, having imposed upon them, however, the absolute responsibility for their own safety and protection as well as for the means of sustenance. On the stock-farm, on the other hand, they are subject to these conditions only in a modified form by way of, to a certain extent, restricted freedom, which is compensated for by the means of protection and oversight as well as a proportion of the means of sustenance regularly provided for them.

3. *Opportunities which Constitute the Basis of Original Observation.* Those whose business brings them into close and frequent contact with farm animals, and who are sufficiently observant, find a large field of opportunities which constitutes the basis for original observation. They cannot fail to observe the evolutionary modifications brought about by any change of environment, the climatic and geographical variations, change of pasture and water; also the imposing characteristic traits of heredity, atavism, temperament, etc. The sum total of these opportunities and observations also constitute the school from which the intelligent stock-owner and breeder learns his first and probably best lessons, and from which he soon learns to distinguish the differences between or departures from a normal healthy condition to that state which is characterized as sickness or disease. The peculiar nature of the sickness or disease may and generally does outrange his limit of comprehension, but the manifest symptoms which distinguish the unhealthy from the healthy condition are always more or less apparent to him.

4. *Non-professional Stock-farm Veterinary Practice.* To the conditions referred to above are also traceable the great inducements which a large number of stock-owners and breeders seemingly appreciate as falling within their sphere of duty, to enter the arena of pathology, therapeutics, etc., under the guise of "non-professional." In a vast majority of cases the results of such ventures are, perhaps, only too well known, and the bitterness of the experience not soon forgotten by the venturer.

5. *Opportunities for Observation by the Professional Student.* All the opportunities which present themselves to the mind of the stock-farm owner and breeder are, with still greater possibility, open to the professional student who is capable of utilizing his powers for observation in a systematic and regular manner, or, in a single word, scientifically. From a previous knowledge of the several scientific theories relating to those conditions for which the stock-farm offers the very best field for practical observation he soon realizes that the seemingly tedious and unpopular, and, to some extent, useless and burdensome theoretical ideas of the class-room and lecture-room reduce themselves to practical applicability with a charming exactness. He appreciates how nature, with a subtle plasticity, gradually and with definite purpose moulds and develops into the most graceful adult forms the seemingly ill-shaped and ill-proportioned young. The watchful care and concern of the natural parent for the young, and her evident pleasure in its being, can nowhere be so fully appreciated as on the stock-farm. When the conditions calling for the practical application of the principles of medicine and surgery arise, the wide range of difference between the environments of the stable in city or town and the barn of the farm at once suggests such modifications as would reduce those principles to their very simplest forms. In the absence of the more complete assortment of the city drug-store the simpler and more ordinary articles of domestic use have very often to be called into requisition, and with the most charming results. In the absence of the elaborate and scrupulously kept operating-table of the city infirmary, a carefully sprinkled cement floor, or continuous antiseptic spray, the carefully regulated light, etc., the stock-farm offers the shade of an overspreading tree, under which bundles of straw may be spread and sprinkled, or even an open lawn covered with sufficient turf, with abundant light from the firmament above, with air sufficiently purified by the sun's rays and warmth as to reduce the number of germs to a minimum. These comparisons refer with directness to the more ordinary cases calling for treatment; but when the more serious conditions, such as epidemic of contagious and infectious animal disease, march in or break out upon the field, being communicated either by diseased conditions existing on neighboring or adjoining farms, or introduced through the medium of new acquisitions to the farm, then the plan of warfare has to be modified and changed to meet the special conditions, and recourse to isolation, quarantine, preventive inoculation, disinfection, etc., becomes imperative, the success or effectiveness from any or all of these methods being just in proportion to the thoroughness with which said methods are carried out and applied.

6. *The Stock-farm as a Natural Experiment Station.* The stock-farm, managed with intelligence and foresight sufficient to appreciate the changes and variations of conditions as they arise, and the spirit to investigate and endeavor to inquire into the causes which lead up to the changed conditions, the ultimate consideration of what would be the best remedy to apply and the particular methods under which the application may be made, such a farm unquestionably offers the most perfect features of an ideal experiment station. If, by recurrence, endemic disease is proven to be permanently established in any particular section of the territory, the particular time of its recurrence, the climatic and atmospheric conditions under which it recurs, the severity with which the periodic outbreaks manifest themselves, the particular grades or species of animals which are more or less susceptible to the invasion, the physical condition which more readily succumbs to the attack, the principal channels or media which facilitate the spread and dissemination of the contagious and infectious principles, and, finally, the agents and methods which are most effectual in the control and stamping out of such disease.

The opportunities for engaging in experimental work of this kind on an extended scale by the simplest and yet most effective methods can be found only on the stock-farm, where the professional student willing and able to grasp and profit by them finds that the lessons there offered him constitute a valuable post-graduate course.

